# Treatment and recurrence of giant cell tumors of bone – A retrospective cohort from a developing country

**DOI:** 10.1016/j.amsu.2019.10.010

**Published:** 2019-10-15

**Authors:** Obada Hasan, Moiz Ali, Mohammad Mustafa, Arif Ali, Masood Umer

**Affiliations:** a Resident Section of Orthopaedics, Department of Surgery, Aga Khan University Hospital Karachi, Pakistan; b Resident Department of Surgery Abbasi Shaheed Hospital, Karachi, Pakistan; c Department of Surgery the Indus Hospital, Karachi, Pakistan; d Section Head Department of Orthopedic Surgery, Aga Khan University Hospital, Karachi, Pakistan

**Keywords:** Giant cell tumor, Recurrence, Survival

## Abstract

**Introduction:**

GCT is a benign primary bone tumor which is known to cause local recurrence as well as distant metastases. The standard care of treatment of GCT in our institution is the extended intralesional curettage followed by the use bone cement and either phenol or alcohol as adjunct therapy. This offers preservation of joint closest to tumor and decreased risk of recurrence compared to curettage alone. Therefore, the objective of this study was to assess the recurrence of GCT of the bone and time of recurrence-free survival after primary surgery (curettage with adjunct therapy) and determine the influence of factors like site of tumor involvement and demographic factors on the risk of recurrence.

**Methods:**

Non-funded, non-commercial single group retrospective cohort study was conducted at a tertiary care university hospital. Total of 44 patients treated for primary GCT of the bone between 1995 and 2015 at our institution were included. Medical record files were reviewed for demographic characteristics, intra-operative findings and post-operative follow-up. Risk factors for recurrence and mean recurrence free survival was calculated using appropriate statistical analysis.

**Results:**

Proximal tibia was the most commonly involved bone followed by distal femur, while intralesional curettage with either phenol or alcohol as adjunct was the most common primary treatment. Mean follow-up period for all patients was 52.1 ± 43.9 months. Out of the 46 tumors operated primarily at our institution, recurrence developed in eight (17.4%) cases. Extra-compartmental spread of tumor and tumor grade were identified to have a significant association with recurrence (P = 0.013 and 0.043 respectively). Estimated recurrence free survival at 2 and 5 - year interval was 0.85 and 0.83 respectively.

**Conclusion:**

Extra-compartmental extension of tumor and a higher-grade lesion is significantly associated with development of recurrence in cases of GCT of bone.

## Highlights

• GCT is a benign primary bone tumor which is known to cause local recurrence as well as distant metastases.
• Aim was to assess the recurrence of GCT of the bone and time of recurrence-free survival after primary surgery.
• Total of 56 patients treated for GCT of the bone between 1995 and 2015 at our institution were included.
• Extra-compartmental extension of tumor and a higher-grade lesion are significantly associated with the tumor recurrence.

## 1 Introduction

Giant cell tumor (GCT) of the bone is one of the rare benign, primary, bone tumors and accounts for around 5% of all primary bone tumors [1]. Most commonly occurring in the metaphyseal region of the long bones, but these tumors can arise in any part of the skeleton [2]. Clinically, GCT ranges from inactive tumors to aggressive tumors with local extension destroying the cortex and involving the surrounding soft tissue. Radiographic images show lytic lesions without significant classification [3].

The standard care of treatment of GCT in our institution is the extended intralesional curettage followed by the use bone cement and either phenol or alcohol as adjunct. Being the least of the invasive surgical methods, it offers the option of saving the joint closest to the tumor. Use of adjuncts has shown to decrease the risk of recurrence compared to curettage alone and hence the procedure is now widely accepted as the treatment of choice in various parts of the world [4,5].

As mentioned, one of the main complications of GCT of bone is local recurrence which hinders the clinical course of treatment. Few of the factors shown to affect recurrence are type of surgery and extra-compartmental extension [2,[5], [6], [7]]. Some authors have reported increased recurrence rates in primary tumors with soft tissue extension [1,6], whereas others have not seen such an effect [8,9]. Even though belonging to the benign type, GCTs are also able to metastasize to the lungs with reported metastases rates varying between 1 and 6% [6,10,11]. Metastasis at time of presentation has also been postulated as a risk factor for recurrence [12,13].

Therefore, the objective of this study was to assess the recurrence of GCT of the bone and time of recurrence-free survival after primary surgery at our institution, and to determine the influence of patient and disease related factors like demographic characteristics and tumor location, soft tissue extension etc. respectively on the risk of recurrence.

## 2 Patients and methods

A non-funded, non-commercial retrospective single group cohort study was conducted at a tertiary care university hospital in a developing country. Research protocol was developed prior to the study start-up and is available from the corresponding author upon request. All patients with GCT who were diagnosed and treated at our institution from 1st January 1995 to 31st December 2015 were included. The included patients had initially undergone radiological imaging including X-ray, computed topography (CT) scan and/or magnetic resonance imaging (MRI) scans to evaluate the radiological features and extent of disease and final diagnosis of GCT in each case was confirmed via biopsy of the tumor conducted in clinic or during surgery.

**Fig. 1:**
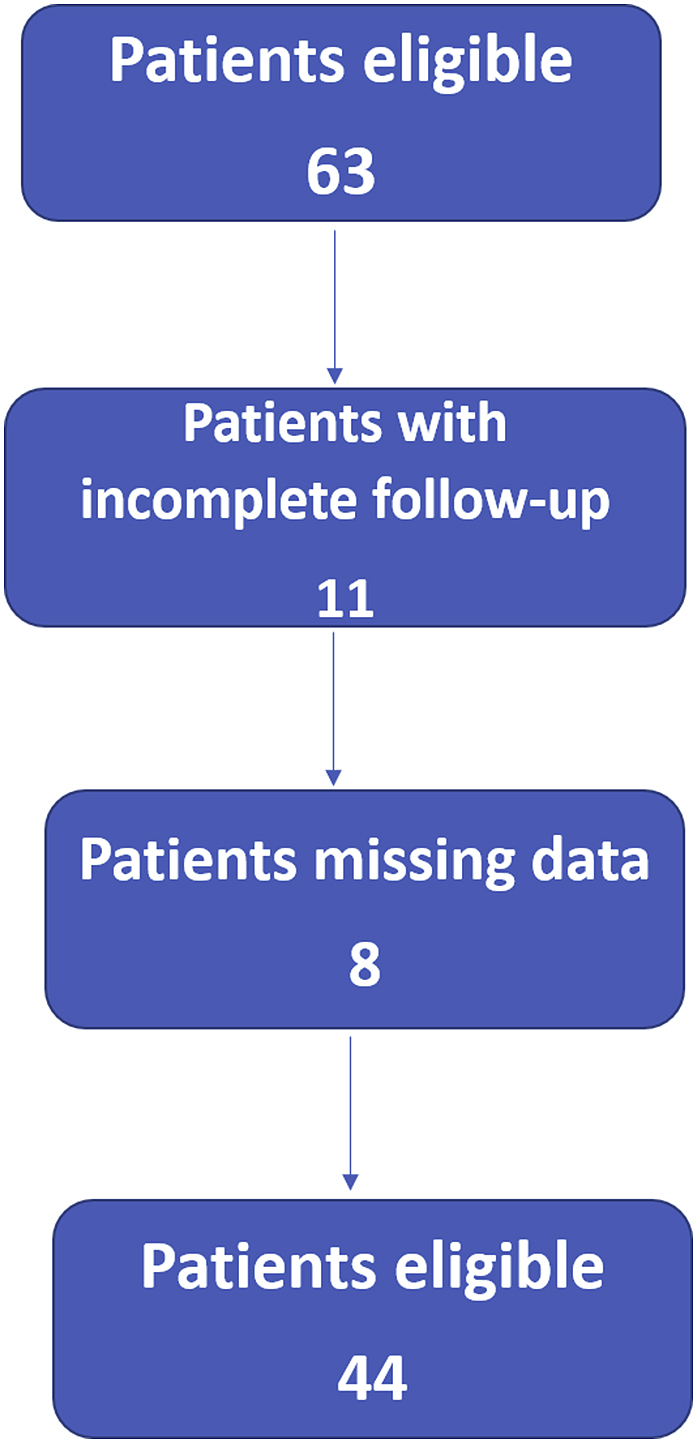
Patient's inclusion flow chart.

In total, there were 63 patients who met the inclusion criteria, however only 44 of these patients were included; rest of the patients either had incomplete follow-ups or missing data in their files (Fig. 1). There were two patients with multiple lesions at time of presentation, hence each lesion was analyzed as a separate tumor. Medical record files of all patients were reviewed for collection of data including any important pre-operative findings e.g. pathological fractures. Tumor growth either intra-compartmental or extra-compartmental was identified based on preoperative imaging studies and intraoperative findings; following which each patient was graded according to the Campanacci Grading System.

**Fig. 2:**
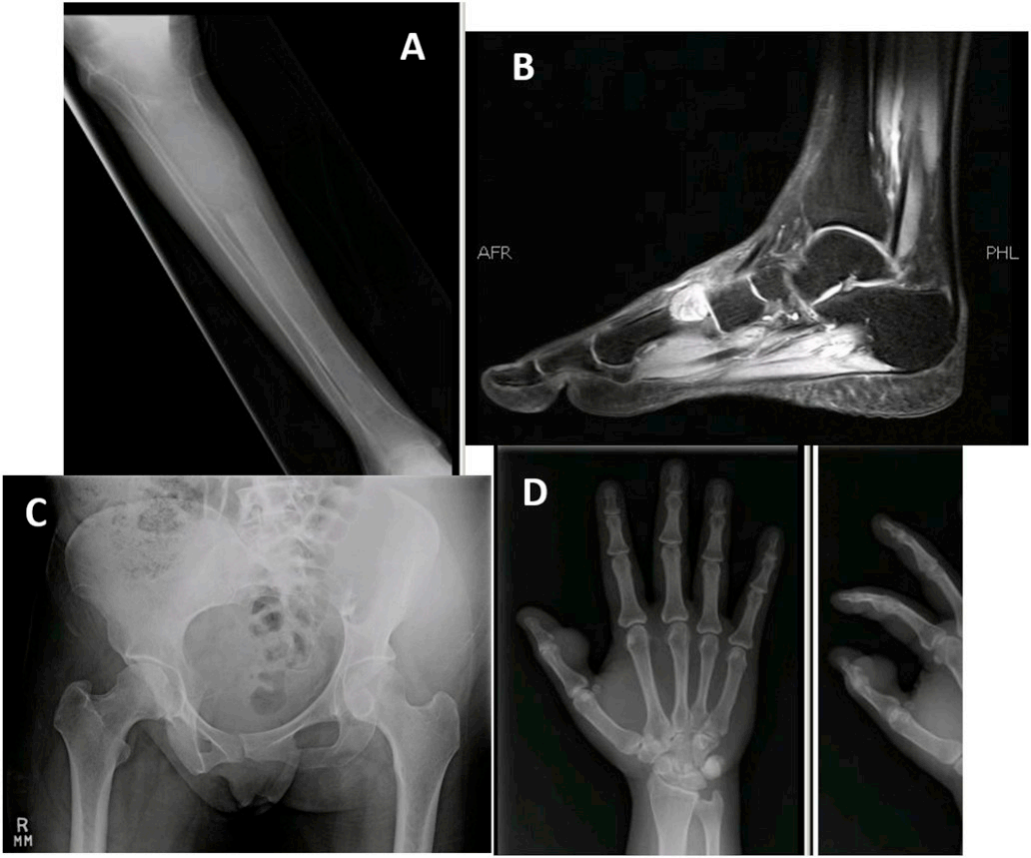
Rare locations and presentations of GCT. A: In tibia, proximal GCT and distal Brown tumor in a patient with primary hyperparathyroidism. B: GCT of first metatarsal base. C: GCT of left iliac blade and sacrum. D: GCT of proximal phalanx of thumb.

**Fig. 3:**
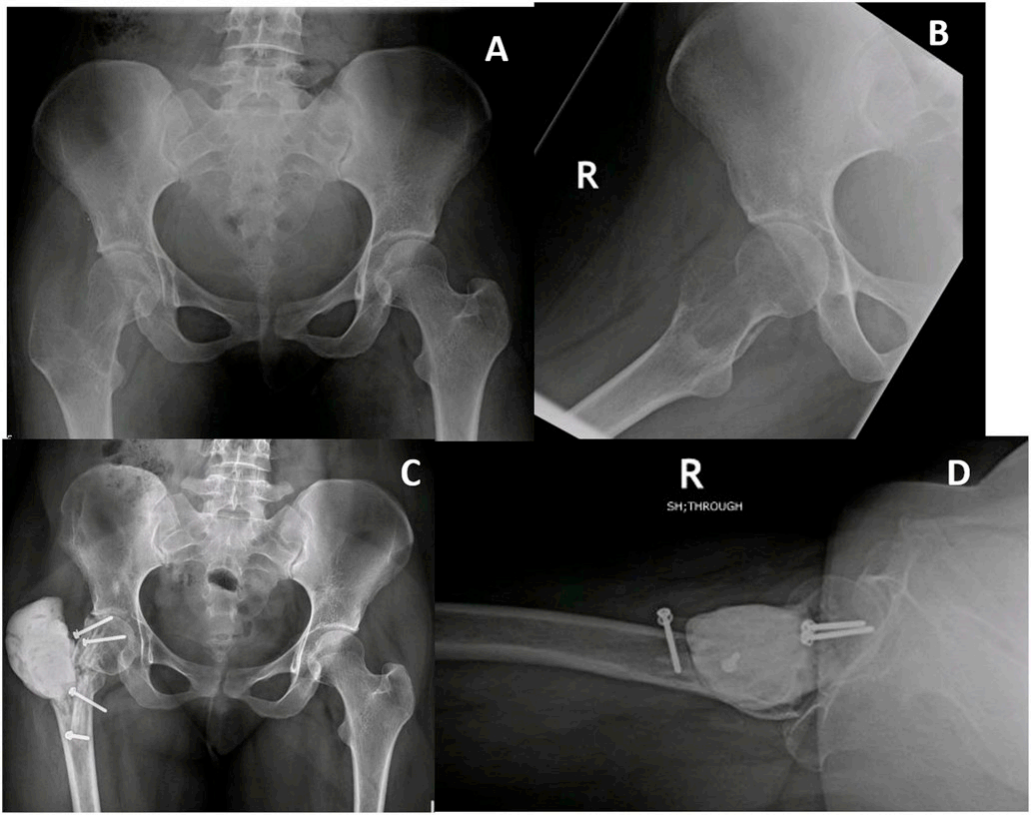
Preoperative (A & B) and postoperative (C & D) X-ray images of GCT proximal femur with salvaging of head of femur.

**Fig. 4:**
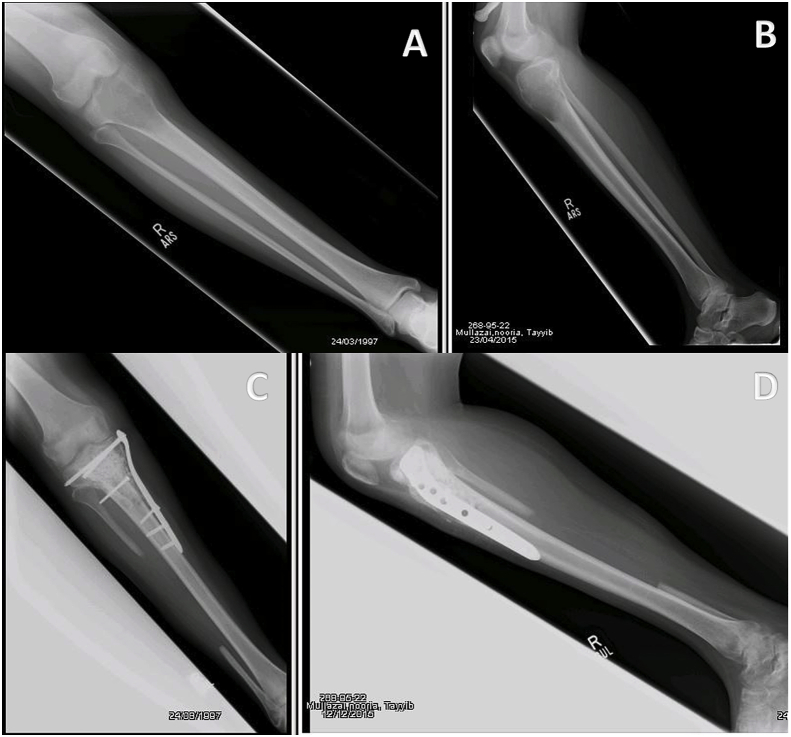
(A & B): Preoperative x-rays of 18 years old girl with knee pain for 4 months showing the typical appearance of GCT in proximal tibia along with soft tissue extension. (C & D): Postoperative x-rays after extended curettage, fibula bone graft, PMMA and stabilization of locking plate.

**Fig. 5:**
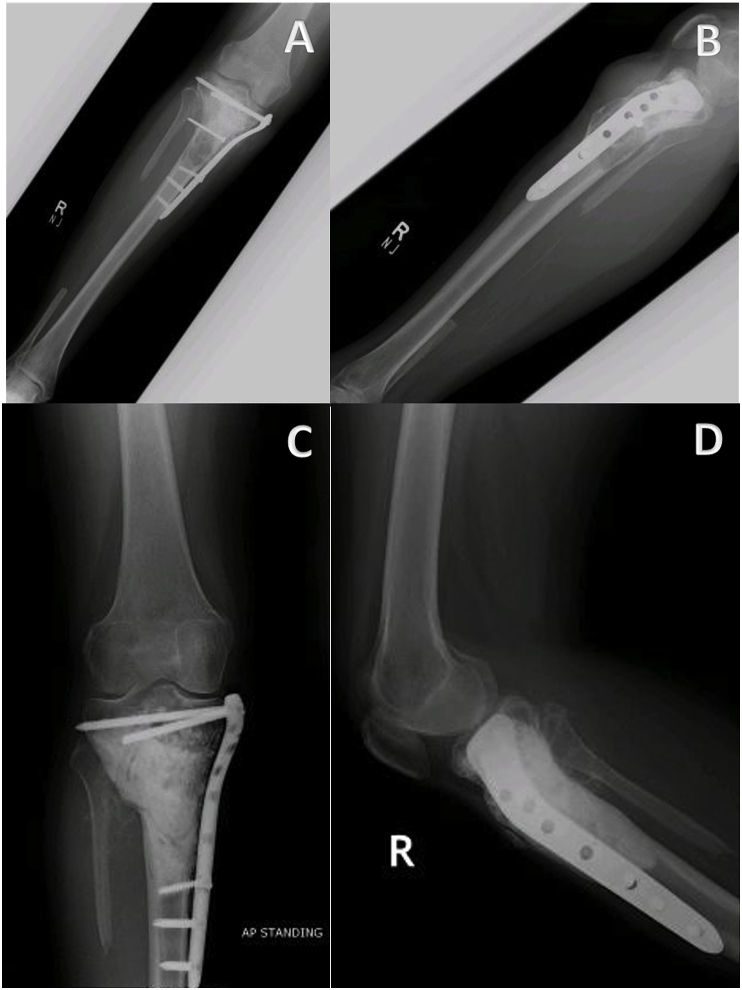
Same patient in Fig. 3 presented after 20 months with history of knee pain for a month. (A & B): Diagnosed as recurrence of GCT. (C & D): Extended curettage and PMMA done.

All patients included in the study were treated before the introduction of Denosumab at our institution, hence the treatment of choice was intralesional curettage with use of bone cement and either phenol or alcohol as adjunct therapy for primary disease. However, patients with tumor on atypical sites including pelvis, spine, metatarsals and hand were treated with wide margin excision. A few X-ray images of tumors in atypical locations are shown in Fig. 2. All surgeries were performed by senior fellow-ship trained orthopedic oncologists with more than five years of experience. Fig. 3, Fig. 4 show pre-operative and post-operative X-ray images of two patients selected randomly while Fig. 5 shows X-rays of the same patient as in Fig. 3 after he presented with recurrence. With regards to post-operative clinical course; recurrence after primary surgery, number of recurrences and treatment of recurrence were recorded. The follow-up protocol consisted of conventional radiography of the primary tumor site at 1.5, 3, and 6 months postoperatively, followed by half-yearly radiographs until two years postoperatively. Data was collected by orthopedic residents who were not part of the operating teams to avoid any observer bias.

Approval for the study was obtained from the Ethical Review Committee of our institution and the study was registered at ClinicalTrials.gov with the assigned Identification number: NCT03854136. The collected data was analyzed using Statistical Package for Social Sciences (SPSS, Inc, Chicago, IL, USA) 23. Results were recorded as a mean ± standard deviation for quantitative variables and as percentages for qualitative variables. Risk factors for recurrence; age, sex, tumor site, soft tissue extension, and pathological fractures, were assessed using Chi-square test. Recurrence free survival was calculated by Kaplan- Meier survival curve. Time to recurrence was defined as time from primary surgery to the date on which a recurrence was confirmed by biopsy. Furthermore, this work has been reported in line with the Strengthening of Cohort Studies in Surgery (STROCCS) criteria [14].

## 3 Results

In all, the total number of patients included was 44 with total of number of tumors which were analyzed being 46. The mean age of presentation was 34.34 ± 12.62 years (range: 16–65 years) and 21 (47.7%) patients were males compared to 23 (52.3%) females. There were two patients with multiple tumors at time of presentation, one of them had lesions at proximal and distal tibia while other had lesions at distal radius and proximal tibia. In both cases, each tumor was analyzed separately.

Table 1 shows frequencies of various tumor related characteristics. The most common location for GCT was found to be proximal tibia with 13 cases, followed by distal femur (10 cases). Majority of the patients (52.2%) had tumor localized to the intra-compartmental region while 47.8% patients had extra-compartmental extension of tumor. Mean follow-up duration for all patients was 52.1 ± 43.9 months (range: 23–206 months). Intralesional curettage was performed in 39 cases as primary treatment, while in 6 cases wide margin excision was performed and hemipelvectomy was done in one case.

**Table 1:** Frequency of tumor characteristics (n = 46).

Characteristics	No. of cases (%)
Pathological fracture at time of diagnosis	
Yes	13 (28.3)
No	33 (71.7)
Site of tumor	
Distal Femur	10 (21.7)
Proximal Femur	5 (10.9)
Distal Tibia	1 (2.2)
Proximal Tibia	13 (28.3)
Humerus	1 (2.2)
Radius	9 (19.6)
Pelvis	2 (4.3)
Lumbar vertebrae	1 (2.2)
Hand	2 (4.3)
Metatarsal	2 (4.3)
Extent of tumor spread	
Intra-compartmental	24 (52.2)
Extra-compartmental	22 (47.8)
Tumor grade (Campanacci classification)	
Grade I	10 (21.7)
Grade II	14 (30.4)
Grade III	22 (47.8)
Procedure performed	
Intralesional curettage with adjunct	39 (84.8)
Wide margin excision	6 (13.0)
Hemipelvectomy	1 (2.2)

Out of the 46 tumors (44 patients) which were primarily operated upon at our institution, recurrence was observed in eight (17.4%) cases. Age, gender, tumor site or associated pathological fracture were not found to be significantly associated with recurrence (Table 2, Table 3). However, extra-compartmental extension of tumor and tumor grade had a significant association with recurrence (P = 0.013 and P = 0.043 respectively). With regards to tumor grade, Grade III tumors were more likely to have recurrence compared to Grade I and II (Table 3). In seven out of eight cases of recurrence, the primary treatment was intralesional curettage while the remaining one patient had undergone wide margin excision.

**Table 2:** Cross tabulation of patient related risk factors with recurrence (n = 44).

Characteristics	Recurrence		P-value
	Yes	No	
Age (years)	33.8	34.5	0.89
Gender			0.89
Male	4 (19.0)	17 (81.0)	
Female	4 (17.4)	19 (82.6)

**Table 3:** Cross tabulation of tumor related risk factors with recurrence (n = 46).

Characteristics	Recurrence		P-value
	Yes	No	
Pathological fracture at time of diagnosis			0.82
Yes	2 (15.4)	11 (84.6)	
No	6 (18.2)	27 (81.8)	
Site of tumor			0.81
Distal Femur	1 (10.0)	9 (90.0)	
Proximal Femur	1 (20.0)	4 (80.0)	
Distal Tibia	0 (0.0)	1 (100)	
Proximal Tibia	4 (30.8)	9 (69.2)	
Humerus	0 (0.0)	1 (100)	
Radius	1 (11.1)	8 (88.9)	
Pelvis	1 (50.0)	1 (50.0)	
Lumbar vertebrae	0 (0.0)	1 (100)	
Hand	0 (0.0)	2 (100)	
Metatarsals	0 (0.0)	2 (100)	
Extent of tumor spread			0.013
Intra-compartmental	1 (4.2)	23 (95.8)	
Extra-compartmental	7 (31.8)	15 (68.2)	
Tumor grade			0.043
Grade I	0 (0)	10 (100)	
Grade II	1 (7.1)	13 (92.9)	
Grade III	7 (31.8)	15 (68.2)	
Procedure performed			0.90
Intralesional curettage with adjunct	7 (17.9)	32 (82.1)	
Wide margin excision	1 (16.7)	5 (83.3)	
Hemipelvectomy	0 (0.0)	1 (100)	

**Fig. 6:**
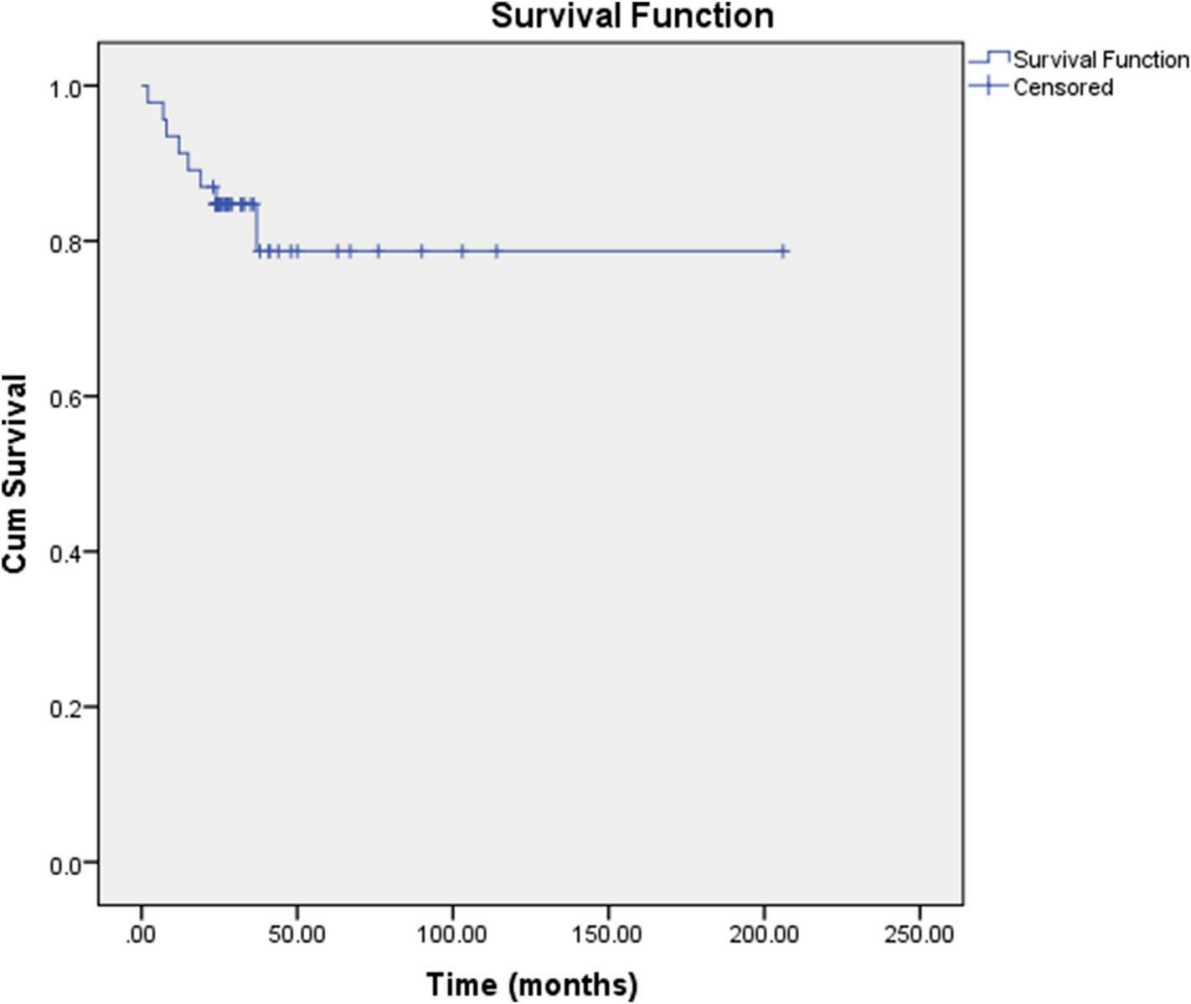
Kaplan-Meier analysis for recurrence free survival.

Fig. 6 shows Kaplan-Meier analysis graph for recurrence free survival for the 46 tumors primarily operated at our institute. At 2-year post-operatively, recurrence free survival was calculated to be 0.85, while at 5-year post-operatively the survival was calculated to be 0.83. The mean time for detection of recurrence was 15.5 ± 11.1 months. All patients with recurrence underwent revision surgery following the same protocol as the primary surgery. One patient presented with pulmonary metastasis with recurrence and hence was referred to oncology after revision surgery. In addition, one patient who had undergone intralesional curettage as primary and revision treatment presented with a second recurrence. He was offered surgery and oncology review but then was lost to follow-up.

## 4 Discussion

Intralesional curettage with adjunct treatment is the most widely practiced procedure for GCT of bone, irrespective of tumor grade and soft tissue extension [15,16]. The treatment of choice in our study was also intralesional curettage using bone cement and either phenol or alcohol as adjunct treatment as it preserves normal bone architecture with minimal post-operative complications [17]. Wide margin excision was only performed where bone salvage was not possible e.g. at lumbar vertebrae, hand and metatarsals. Hemipelvectomy had to be performed in one case of pelvic GCT due to excessive bone involvement and difficult window for curettage.

Proximal tibia and distal femur have been shown to be the most common locations for GCT of bone, as also reported in our study [16,17]. However, in the current study, few tumors were also reported in uncommon locations including two cases of hand and two patients with GCT of metatarsals. With regards to association of tumor location with recurrence, Siddiqui MA et al. showed significantly higher recurrence with proximal tibial involvement [18], while other studies including this one have found no such association [4,15,16].

The overall recurrence rate of 17.4% in our study is comparable to the international literature where recurrence rates have been shown to vary from 15 to 60%. Most of the studies in fact have reported rates of higher than 20%, which places our risk at the lower end of that which is internationally observed [5,16,17,19,20]. Having said that, recurrence rates have been shown to vary considerably with the type of surgery performed. Intralesional curettage without any adjuvant has been associated with highest recurrence rates while wide margin excision has a significantly lower risk for recurrence [4,17]. In the current study, no significant difference was noted in recurrence rates with intralesional curettage compared to wide margin excision, however, due to the small sample size for wide margin excision (n = 8), these results can not be entirely relied upon. In addition, only 1 (1.79%) patient developed distant metastasis which is also comparable to international literature where rate of metastases for GCT has been shown to vary from 1 to 6% [6,10,11]. This patient had a Grade III lesion with extra-compartmental extension which could have contributed to development of his pulmonary metastasis. He was referred to medical oncology for opinion regarding chemoradiation therapy but was lost to follow-up after that.

The two significant risk factors for recurrence identified in the current study include extra-compartmental (soft tissue) extension and tumor grade, similar to previously reported literature. A study conducted in Netherlands in 2012 showed significantly higher recurrence rates of GCT of bone with soft tissue extension (P = <0.001) [15]. Another study by Balke et al. in Germany also showed a significant association of soft tissue extension with recurrence of GCT, with recurrence rates being 29.7 and 16.2% for tumors with and without soft tissue involvement respectively (P = 0.045). In their study, higher rate of recurrence was also noted with Grade III tumors (31.3%) compared to Grade II tumors (20%), although the difference was not shown to be statistically significant [5].

The overall recurrence free survival for primary GCT of bone has also been shown to vary considerably with regards to the type of procedure. Knochentumoren A et al., in 2008 reported an overall 10-year recurrence free survival of 0.74; but survival with wide margin excision was reported to be 0.98 compared to 0.67 for intralesional curettage. 10-year survival specifically for intralesional curettage with bone cement and phenol was 0.73 [4]. Another study by van der Heijden L et al. consisting of 93 patients with primary GCT reported 2 and 5-year recurrence free survival of 0.84 and 0.72 respectively. More recently, Siddiqui MA et al. from Singapore reported a recurrence free survival of 0.86, 0.79 and 0.72 for years 1, 2 and 3 respectively [18]. The 2 and 5-year recurrence free survival rates of 0.85 and 0.83 in the current study are therefore comparable and in fact on the higher end of that shown in international literature when considering majority of the surgeries were intralesional curettage. For GCT of bone, a recurrence after three years is very rare, hence, it can be safely assumed that a 5-year recurrence free survival estimate is a good indicator for further long term survival as well [17].

In addition to the 46 cases primarily operated upon at our institution, 12 patients had also presented to us with recurrence of bone GCT during this time period who underwent primary surgery at another hospital. They were also offered intralesional curettage with adjunct therapy for revision surgery and were followed for development of second recurrence which was observed in one patient. Compared with recurrence of primary GCT of bone, studies have in fact reported a higher rate of recurrence of recurrent bone GCT. In a study conducted in Canada by Turcotte RE et al., a recurrence rate of 10% was observed with primary tumors compared to 35% with recurrent GCT [21]. Similarly van der Heijden L et al. also reported a recurrence rate of 26.9% with primary tumor against 46.7% with recurrent tumor. The 2 and 5-year recurrence free survival was also considerably lower for recurrent tumors compared to primary [15]. In contrast to previously reported re-recurrence rates, out of a total of 20 patients with recurrent disease (including the patients presenting to us with recurrent disease), two (10%) developed a second recurrence compared to a recurrence rate of 17.4% for primary disease.

Although intralesional curettage is associated with higher rates of recurrence compared to wide margin excision, risk of recurrence with curettage also depends on the expertise of the operating surgeon and how well he is able to identify and access pockets of residual disease [17]. Due to financial constraints and lack of resources in our part of the world, we still have to rely on less aggressive treatment options including intralesional curettage with adjunct treatments. However, the comparatively lower recurrence rates reported in the current study with majority of the patients undergoing intralesional curettage, in effect represent the skills of the operating surgeons. It is also important to note that this orthopedic oncology centre was developed in a resource constraint setting to provide quality care to patients suffering from orthopedic tumors where there is considerable lack of awareness and appropriate centres for treatment. Therefore, the results of this study further substantiate the role and effectiveness of the orthopedic oncology centre in question in providing quality care to its patients which is comparable to other parts of the world.

The current study design provided us with the advantage of selecting a cohort group with specific characteristics to meet the objective of our study and hence provide evidence regarding effectiveness of our orthopedic oncology unit. Having said that, the retrospective nature of the study is one of its limitations which may have resulted in information bias. The small sample size also makes it difficult to draw substantial conclusions based on the results. However, to the best of our knowledge, no such study has been published from a developing country such as Pakistan, where resource constraints and lack of awareness make it extremely difficult to effectively manage patients, specifically those of orthopedic oncology. In such a setting, the results of this study are quite promising and show the possibility of achieving standard quality of care even in developing countries. Given that all tumors were operated upon by experienced and fellowship trained orthopedic oncologists at the same centre, recurrence of tumor can be attributed to factors other than surgical technique e.g. tumor spread and grade, as already discussed. We still recommend large scale multi-centre prospective studies with adequate comparison groups to further substantiate the findings.

## 5 Conclusion

Based on the results of the current study, we conclude that soft tissue extension and higher tumor grades are significantly associated with development of recurrence in cases of bone GCT. However, due to low proportion of patients undergoing wide margin excision, association of recurrence risk with type of procedure can not be reliably deduced.

## Ethical approval

Study was approved by the Ethical review committee of the Aga Khan University Hospital Karachi Pakistan.

ERC number: 4687-Sur-ERC-17.

## Sources of funding

There is no funding source related to this article.

## Author contribution

**Obada Hasan:** Study design, analysis, writing of first draft, final review and approval.

**Moiz Ali:** editing, methodology and final review and approval.

**Mohammad Mustafa:** design, editing and writing of the manuscript, final review and approval.

**Arif Ali:** Design, manuscript writing final review and approval.

**Masood Umer:** design and methodology, overall supervision of the paper, final review and approval.

All authors are in agreement for submission of this article in Annals of Medicine and Surgery.

## Registration of research studies

Name of the registry: clinicalTrials.gov.

Unique Identifying number or registration ID: NCT03854136.

Hyperlink to the registration (must be publicly accessible):

## Guarantor

Dr Obada Hasan.

Dr Masood Umer.

## Consent

Patient confidentiality measures were strictly followed.

## Assistance with the study

None.

## Financial support and sponsorship

None.

## Presentation

None.

## Provenance and peer review

Not commissioned, externally peer reviewed.

## Declaration of competing interest

None.
